# Analysis of the clinical features of chest pain in adolescents and children in the Shihezi area of Xinjiang

**DOI:** 10.3389/fped.2026.1890242

**Published:** 2026-07-27

**Authors:** Yan Guo, Hu Li, Lin Liu, Yonglin Chen, Li Zhang, Muqing Niu, Fengling Zhang, Longlong Zhu, Xueting Guo, Jinyong Pan

**Affiliations:** 1 The First Affiliated Hospital of Shihezi University, Shihezi, Xinjiang, China; 2 Department of Pediatrics, The First Affiliated Hospital of Shihezi University, Shihezi, Xinjiang, China

**Keywords:** adolescents, chest pain, clinical characteristics, etiological analysis, Shihezi, Xinjiang

## Abstract

**Objective:**

To investigate the causes and clinical characteristics of chest pain in adolescents aged 8-17 years in the Shihezi area of Xinjiang, and to provide reference for clinical diagnosis and treatment.

**Methods:**

A retrospective analysis was conducted on 243 children aged 8-17 years who presented with non-traumatic chest pain at the Pediatric Emergency and Outpatient Department of the First Affiliated Hospital of Shihezi University from January 2023 to December 2024. Clinical data, including medical history, physical examination findings, laboratory tests, and imaging studies, were collected. Continuous variables were compared using one-way analysis of variance or the Kruskal–Wallis test, and categorical variables were compared using the chi-square test or Fisher's exact test, as appropriate. An exploratory multivariable logistic regression analysis was performed to assess factors associated with cardiac chest pain classification.

**Results:**

Non-cardiac causes were the most common reason for chest pain (47.7%), followed by cardiac causes (20.6%), while the cause remained undetermined in 31.7% of the cases. Work-rest relationship, NRS score, shortness of breath, and palpitations differed significantly among the three groups, whereas chest pain duration, syncope, and fatigue did not. Sex distribution showed a borderline difference among groups (*P* = 0.050). Myocardial biomarkers were higher in the cardiac group; however, these findings should be interpreted cautiously because these biomarkers may have contributed to etiological classification. The non-cardiac group showed the highest abnormal rates in chest x-ray and pulmonary function tests. In exploratory multivariable analysis, NLR, HDL-C, CRP, and LDH were associated with cardiac chest pain classification, but the model was limited by a low events-per-variable ratio.

**Conclusion:**

Clinicians should emphasize detailed history taking and physical examination and select auxiliary tests according to clinical risk features. Chest x-ray and pulmonary function abnormalities were more frequent in patients with respiratory diagnoses, whereas abnormal electrocardiography and elevated myocardial biomarkers were more common in patients classified as having cardiac chest pain. However, these findings indicate associations rather than diagnostic accuracy.

## 1 Introduction

Chest pain is a common complaint during childhood, and parents frequently visit pediatric emergency and outpatient departments out of concern for potential serious heart problems in their children (1). Although chest pain caused by complex cardiac diseases in children is challenging to diagnose and treat, such conditions remain a central concern for parents (2). Overly worrying about chest pain may increase children's anxiety, thereby exacerbating the pain symptoms (3). Previous studies have shown that pediatric chest pain is usually non-cardiac in origin, with respiratory, gastrointestinal, psychological, musculoskeletal, and idiopathic causes being common. However, data from the Shihezi area of Xinjiang remain limited. Routine electrocardiograms and chest x-rays may sometimes fail to identify the cause, requiring further tests such as echocardiography and ambulatory electrocardiography to assess for possible cardiac conditions (4). Therefore, detailed history taking, physical examination, and targeted auxiliary tests are essential components in the diagnosis and treatment of chest pain in children (5). The etiological distribution of pediatric chest pain may vary according to study setting, referral pattern, diagnostic strategy, and local healthcare practice (6). This study aims to analyze the distribution of causes and clinical features of chest pain in adolescents and children in the Shihezi area of Xinjiang, providing a basis for improving local diagnostic and therapeutic levels.

## 2 Materials and methods

### 2.1 Data source

A total of 243 non-traumatic chest pain patients aged 8-17 years who visited the Pediatric Emergency and Outpatient Department of the First Affiliated Hospital of Shihezi University from January 2023 to December 2024 were selected. Data on their medical history, physical examination, laboratory tests (such as complete blood count, inflammatory markers, myocardial biomarkers, etc.), and imaging studies (such as chest x-ray, electrocardiogram, echocardiogram, coronary CTA, etc.) were collected. Exclusion criteria were: (1) traumatic chest pain patients; (2) patients younger than 8 years or older than 17 years.

### 2.2 Data collection

A retrospective review of the clinical data from 243 patients was conducted: the average age was 12.61 ± 4.38 years, with 87 male patients (35.8%) and 156 female patients (64.2%). The average weight was 51.26 ± 12.72 kg, and the average BMI was 20.83 ± 3.17 kg/m^2^. Chest pain intensity was recorded using the Numerical Rating Scale (NRS), an 11-point scale (0 = no pain, 10 = worst possible pain). A clear etiology was identified in 166 cases, which included:

Respiratory system diseases (74 cases): lobar pneumonia (38 cases), bronchial asthma (25 cases), allergic asthma (7 cases), tuberculosis (2 cases), spontaneous pneumothorax (2 cases).

Psychological disorders (18 cases): anxiety (7 cases), depression (11 cases).

Gastrointestinal diseases (15 cases): chronic gastritis (6 cases), esophagitis (3 cases), gastroesophageal reflux disease (6 cases).

Cardiac diseases (50 cases): coronary artery myocardial bridging (17 cases), large atrial septal defect (8 cases), supraventricular tachycardia (6 cases), ventricular septal defect (3 cases), idiopathic pulmonary hypertension (3 cases), myocarditis (3 cases), large pericardial effusion (2 cases), hypertrophic cardiomyopathy (2 cases), severe aortic insufficiency with bicuspid aortic valve malformation (2 cases), coronary artery-to-pulmonary artery fistula (2 cases), coronary artery dilatation with right ventricular fistula (1 case), mitral valve prolapse with severe regurgitation (1 case).

Other systemic diseases (9 cases): gallbladder stones (1 case), acute cholecystitis (3 cases), kidney stones (5 cases).

The cause of chest pain remained unclear in 77 cases.

### 2.3 Research grouping

The children were divided into three groups based on the etiology of their chest pain:

Cardiac chest pain group (50 cases, 20.6%).

Non-cardiac chest pain group (116 cases, 47.7%), including cases with respiratory, psychological, gastrointestinal, and other systemic diseases as described above.

Unknown etiology chest pain group (77 cases, 31.7%).

Demographic indicators, chest pain characteristics, accompanying symptoms, and examination results were compared among the three groups.

### 2.4 Diagnostic criteria for etiological classification

Cardiac chest pain was defined as chest pain considered to be directly attributable to a cardiac disorder based on clinical symptoms, physical examination, and objective cardiac evidence. Cardiac causes included myocarditis, pericardial disease, clinically significant arrhythmia, cardiomyopathy, pulmonary hypertension, severe valvular disease, hemodynamically significant congenital heart disease, and coronary abnormalities with evidence of ischemia or symptom correlation.

Myocardial bridging was classified as a cardiac cause only when it was accompanied by exertional chest pain, ischemic electrocardiographic changes, abnormal exercise electrocardiography, elevated myocardial biomarkers, or pediatric cardiology assessment supporting clinical relevance. Isolated myocardial bridging detected by coronary CTA without objective evidence of ischemia was considered an associated finding rather than a definite cause of chest pain.

Atrial septal defect or ventricular septal defect was classified as a cardiac cause only when there was evidence of hemodynamic significance, such as right heart enlargement, pulmonary hypertension, ventricular dysfunction, significant shunt, exercise intolerance, or specialist assessment supporting symptom attribution. Simple congenital heart defects without these findings were not automatically considered the direct cause of chest pain.

Non-cardiac chest pain included respiratory, gastrointestinal, psychological, urinary, biliary, musculoskeletal, and other non-cardiac diseases considered to explain the symptom. Chest pain was classified as undetermined when no definite etiology could be established after review of available clinical data.

### 2.5 Coronary CTA indications and principles

Coronary CTA was not used as a routine screening examination. It was performed selectively in patients with high clinical suspicion for coronary artery anomalies based on exertional chest pain, high NRS score, abnormal electrocardiographic findings, elevated cardiac biomarkers, abnormal echocardiographic findings, or persistent clinical concern after initial cardiac evaluation. Informed consent was obtained from parents or guardians before coronary CTA. Age-appropriate dose-reduction protocols were used to minimize radiation exposure.

### 2.6 Statistical analysis

Statistical analysis was performed using SPSS version 21.0. Continuous variables were assessed for normality using the Shapiro–Wilk test and visual inspection of histograms. Normally distributed variables were expressed as mean ± standard deviation and compared among the three groups using one-way analysis of variance. Non-normally distributed variables were expressed as median and interquartile range and compared using the Kruskal–Wallis test. Categorical variables were expressed as n/N (%) and compared using the chi-square test or Fisher's exact test, as appropriate.

When overall differences were statistically significant, *post-hoc* pairwise comparisons were performed with Bonferroni correction. Missing data were not imputed, and the actual number of available observations was reported for each variable.

Because this was a retrospective exploratory study and several diagnostic tests were also used in etiological classification, the results were interpreted as associations rather than independent predictors or diagnostic accuracy estimates. A two-sided P value <0.05 was considered statistically significant.

An exploratory multivariable logistic regression analysis was performed with cardiac chest pain classification as the dependent variable and non-cardiac plus unknown etiology chest pain as the reference category. Variables were selected based on clinical relevance and univariate findings, while myocardial biomarkers used in etiological classification were excluded to reduce incorporation bias. Complete-case analysis was used. Model discrimination was assessed using the area under the receiver operating characteristic curve, and calibration was assessed using the Hosmer-Lemeshow test. Because the events-per-variable ratio was below 10, the model was considered exploratory.

## 3 Results

### 3.1 General clinical data

There were no statistically significant differences among the three groups in terms of age, weight, BMI, family history (heart disease/sudden death), resting blood pressure, and heart rate (*P* > 0.05). Sex distribution showed a borderline difference among the three groups (*P* = 0.050), with a higher proportion of females in the unknown etiology group (74.0%), while the non-cardiac chest pain group had the highest proportion of males (43.1%).

The distribution of causes showed that among the non-cardiac chest pain cases, respiratory system diseases were the most common (63.8%). Among the cardiac chest pain cases, coronary artery myocardial bridging was the most frequent (34.0%, 17/50), followed by large atrial septal defect (16.0%, 8/50) and supraventricular tachycardia (12.0%, 6/50).

### 3.2 Clinical characteristics comparison among the three groups

#### 3.2.1 Chest pain characteristics

Duration: The cardiac chest pain group had the highest proportion of chest pain lasting more than 10 minutes (57.1%), but the difference among the three groups did not reach statistical significance (*P* = 0.099).

Pain Score: The cardiac chest pain group had a significantly higher pain score (6.8 ± 2.1) compared to the non-cardiac group (4.2 ± 1.8) and the unknown etiology group (4.5 ± 1.9) (*P* < 0.05).

Work-Rest Relationship: The majority of cases in the cardiac chest pain group experienced pain during exertion (56.0%, 28/50), while the non-cardiac group and unknown etiology group mainly experienced pain at rest (61.9%, 58.4%). The differences among the three groups were statistically significant (*P* < 0.001).

#### 3.2.2 Accompanying symptoms

The cardiac chest pain group had a significantly higher incidence of shortness of breath (36.0%) and palpitations (52.0%) compared to the other two groups (*P* < 0.05).

The comparison of fainting and fatigue rates among the three groups showed no statistically significant differences (*P* > 0.05). (see Table 1).

**Table 1:** Demographic and clinical characteristics of patients according to etiological group.

Item	Cardiac chest pain group (*n* = 50)	Non-cardiac chest pain group (*n* = 116)	Unknown etiology group (*n* = 77)	Statistic	P
Age (years)	11.91 ± 3.92	12.64 ± 4.09	13.02 ± 4.42	1.08	0.341
*Gender (cases)*					0.050
Male	17 (34.0)	50 (43.1)	20 (26.0)	-	-
Female	33 (66.0)	66 (56.9)	57 (74.0)	-	-
Weight (kg)	50.45 ± 13.82	51.92 ± 11.21	50.80 ± 13.28	0.32	0.725
BMI (kg/m^2^)	20.52 ± 2.76	20.96 ± 3.30	20.83 ± 2.83	0.36	0.701
*Family History (cases)*					0.083
Yes	7 (14.0)	6 (5.2)	10 (13.0)	-	-
No	43 (86.0)	110 (94.8)	67 (87.0)	-	-
Systolic BP (mmHg)	118.5 ± 10.2	115.3 ± 9.8	116.7 ± 10.1	1.25	0.289
Diastolic BP (mmHg)	72.3 ± 8.5	70.8 ± 7.9	71.5 ± 8.2	0.98	0.376
Heart Rate (bpm)	82.5 ± 11.3	78.6 ± 10.5	80.2 ± 10.8	1.56	0.213
*Chest Pain Duration*					0.099
<1 minute	4/49 (8.2)	18/116 (15.5)	21/77 (27.3)	-	-
1-10 minutes	17/49 (34.7)	50/116 (43.1)	30/77 (39.0)	-	-
>10 minutes	28/49 (57.1)	48/116 (41.4)	26/77 (33.8)	-	-
Chest Pain Score (NRS)	6.8 ± 2.1	4.2 ± 1.8	4.5 ± 1.9	31.39	<0.001
*Work-Rest Relationship*					<0.001
Exertion	28/50 (56.0)	23/116 (19.8)	18/77 (23.4)	-	-
Rest	12/50 (24.0)	72/116 (62.1)	45/77 (58.4)	-	-
Both	10/50 (20.0)	21/116 (18.1)	14/77 (18.2)	-	-
*Accompanying Symptoms*					
Shortness of breath	18/50 (36.0)	24/116 (20.7)	14/77 (18.2)	-	0.047
Palpitations	26/50 (52.0)	22/116 (19.0)	16/77 (20.8)	-	<0.001
Fainting	5/50 (10.0)	4/116 (3.4)	3/77 (3.9)	-	0.178
Fatigue	16/50 (32.0)	30/116 (25.9)	22/77 (28.6)	-	0.714

Data are presented as mean ± SD or n/N (%). One-way ANOVA was used for normally distributed continuous variables, and the chi-square test or Fisher's exact test was used for categorical variables. Duration of chest pain was missing for 1 patient in the cardiac chest pain group. NRS was missing for 2 patients in the cardiac group, 1 in the non-cardiac group, and 1 in the unknown etiology group.

### 3.3 Laboratory findings

Laboratory findings are summarized in Table 2. Because not all laboratory tests were performed in every patient, the number of available observations differed across indicators. Several laboratory variables were non-normally distributed and are therefore presented as median and interquartile range.

**Table 2:** Laboratory test results according to etiological group, with distribution assessment and *post-hoc* comparisons.

Item	Cardiac group	Non-cardiac group	Unknown group	Distribution	Statistic	P	Post-hoc
WBC	8.9 (6.9-10.0)	7.1 (5.8-8.7)	7.6 (6.8-9.0)	Non-normal	H = 13.82	<0.001	CvsNC*, CvsU, NCvsU*
NEU	4.9 (3.8-6.3)	3.3 (2.6-4.4)	4.0 (3.4-5.1)	Non-normal	H = 35.42	<0.001	CvsNC*, CvsU*, NCvsU*
NLR	2.4 (1.7-3.2)	1.4 (1.0-1.8)	1.6 (1.4-2.1)	Non-normal	H = 42.87	<0.001	CvsNC*, CvsU*, NCvsU*
PLT	219.8 (155.6-265.4)	213.1 (150.5-279.2)	222.2 (171.2-260.8)	Non-normal	H = 0.50	0.777	—
HGB	13.8 (12.8-14.9)	13.9 (12.8-15.4)	13.2 (12.2-14.4)	Non-normal	H = 5.28	0.072	—
PCV	40.4 ± 6.2	41.7 ± 5.5	40.0 ± 5.5	Normal	F = 2.00	0.138	—
RDW	13.1 ± 1.5	13.4 ± 1.9	13.1 ± 1.9	Normal	F = 0.52	0.594	—
MPV	8.8 ± 1.4	8.9 ± 1.3	9.0 ± 1.3	Normal	F = 0.31	0.735	—
ESR	7.2 (4.0-13.1)	6.9 (2.5-13.8)	7.8 (2.2-14.2)	Non-normal	H = 0.10	0.949	—
LDL-C	2.7 (2.5-3.2)	2.3 (2.0-2.8)	2.6 (2.2-3.0)	Non-normal	H = 15.39	<0.001	CvsNC*, CvsU, NCvsU*
HDL-C	1.0 (0.9-1.2)	1.3 (1.2-1.6)	1.2 (1.0-1.4)	Non-normal	H = 28.99	<0.001	CvsNC*, CvsU*, NCvsU
TC	4.6 ± 0.7	4.4 ± 0.7	4.5 ± 0.8	Normal	F = 1.59	0.206	—
TG	1.3 (0.9-1.7)	1.2 (0.9-1.4)	1.1 (0.9-1.5)	Non-normal	H = 2.62	0.270	—
AST	26.4 (20.7-33.4)	20.5 (15.3-28.8)	24.4 (17.8-38.4)	Non-normal	H = 10.76	0.005	CvsNC*, CvsU, NCvsU
ALT	22.8 (16.8-37.1)	25.4 (18.2-37.8)	27.8 (18.5-37.9)	Non-normal	H = 1.14	0.567	—
ALB	38.2 ± 6.1	40.1 ± 5.6	40.3 ± 5.7	Normal	F = 2.10	0.125	—
CRP	12.1 (4.0-20.0)	3.4 (1.8-6.7)	6.0 (2.6-9.4)	Non-normal	H = 23.57	<0.001	CvsNC*, CvsU*, NCvsU*
PCT	0.2 (0.1-0.3)	0.1 (0.0-0.1)	0.1 (0.0-0.2)	Non-normal	H = 23.82	<0.001	CvsNC*, CvsU, NCvsU
hs-cTnI	0.3 (0.1-0.4)	0.0 (0.0-0.0)	0.1 (0.0-0.3)	Non-normal	H = 80.68	<0.001	CvsNC*, CvsU*, NCvsU*
LDH	253.1 (218.1-289.6)	176.5 (156.9-201.6)	212.1 (181.6-244.6)	Non-normal	H = 73.13	<0.001	CvsNC*, CvsU*, NCvsU*
CK	132.4 (90.9-241.0)	66.8 (53.8-81.5)	86.5 (61.8-102.7)	Non-normal	H = 49.89	<0.001	CvsNC*, CvsU*, NCvsU*
CK-MB	6.7 (5.2-14.0)	1.5 (1.0-1.9)	3.0 (2.1-4.5)	Non-normal	H = 136.31	<0.001	CvsNC*, CvsU*, NCvsU*

Data are presented as mean ± SD for normally distributed variables or median (interquartile range) for non-normally distributed variables. The number in parentheses indicates the number of patients with available data. Normality was assessed using the Shapiro–Wilk test. Normally distributed variables were compared using one-way ANOVA, and non-normally distributed variables were compared using the Kruskal–Wallis test. *post-hoc* pairwise comparisons were performed with Bonferroni correction. C, cardiac group; NC, non-cardiac group; U, unknown etiology group. *P < 0.017 after Bonferroni correction. CK, CK-MB, and hs-cTnI may have been used during etiological classification; therefore, comparisons involving these markers are subject to potential incorporation bias.

WBC, NEU, NLR, LDL-C, HDL-C, AST, CRP, PCT, hs-cTnI, LDH, CK, and CK-MB differed significantly among the three groups. Compared with the non-cardiac group, the cardiac group had higher levels of WBC, NEU, NLR, CRP, PCT, hs-cTnI, LDH, CK, and CK-MB. However, myocardial biomarkers may have contributed to the etiological classification of cardiac chest pain; therefore, comparisons involving hs-cTnI, CK, and CK-MB should be interpreted with caution because of potential incorporation bias.

PLT, HGB, PCV, RDW, MPV, ESR, TC, TG, ALT, and ALB did not differ significantly among the three groups.

### 3.4 Imaging and functional test findings

Diagnostic test utilization and abnormal rates are shown in Table 3. Resting ECG abnormalities were more frequent in the cardiac group than in the non-cardiac and unknown etiology groups (70.0% vs. 24.1% and 19.5%, *P* < 0.001). Exercise ECG abnormalities also differed significantly among the three groups (84.4% vs. 46.9% and 32.0%, *P* < 0.001). Dynamic ECG abnormality rates did not reach statistical significance (*P* = 0.069).

**Table 3:** Diagnostic test utilization and abnormal rates by etiological group.

Item	Cardiac group	Non-cardiac group	Unknown group	P
Resting ECG abnormal	35/50 (70.0)	28/116 (24.1)	15/77 (19.5)	<0.001
Dynamic ECG abnormal	32/48 (66.7)	22/40 (55.0)	12/30 (40.0)	0.069
Exercise ECG abnormal	38/45 (84.4)	15/32 (46.9)	8/25 (32.0)	<0.001
Echo: Structural abnormality	38/48 (79.2)	12/35 (34.3)	5/30 (16.7)	<0.001
Echo: EF <50%	8/48 (16.7)	2/35 (5.7)	1/30 (3.3)	0.097
Chest x-ray abnormal	5/50 (10.0)	62/116 (53.4)	12/77 (15.6)	<0.001
Pulmonary function test abnormal	2/10 (20.0)	49/80 (61.3)	8/20 (40.0)	0.019
Coronary CTA abnormal	30/42 (71.4)	5/8 (62.5)	3/7 (42.9)	0.320
Coronary artery myocardial bridging	17/42 (40.5)	0/8 (0.0)	0/7 (0.0)	0.013

Data are presented as abnormal/number of patients who underwent the test (%). The denominator for each item reflects the actual number of patients who underwent that examination rather than the total group size. Categorical variables were compared using the chi-square test or Fisher's exact test, as appropriate. Coronary CTA was performed selectively according to clinical indications and was not used as a routine screening examination.

Echocardiographic structural abnormalities were more common in the cardiac group (79.2%) than in the non-cardiac group (34.3%) and unknown etiology group (16.7%) (*P* < 0.001). The frequency of EF < 50% did not differ significantly among groups (*P* = 0.097).

Chest x-ray abnormalities were most frequent in the non-cardiac group (53.4%), mainly reflecting respiratory diseases, and differed significantly among groups (*P* < 0.001). Pulmonary function test abnormalities also differed among groups and were most common in the non-cardiac group (61.3%, *P* = 0.019).

Coronary CTA was performed selectively in 42 cardiac patients, 8 non-cardiac patients, and 7 patients with unknown etiology. The overall CTA abnormality rate did not differ significantly among groups (*P* = 0.320). Myocardial bridging was detected only in the cardiac group (17/42, 40.5%; *P* = 0.013).

### 3.5 Exploratory multivariable logistic regression analysis

Multivariable logistic regression was performed to explore factors associated with cardiac chest pain classification after adjustment for selected covariates. Because myocardial biomarkers (hs-cTnI, CK, and CK-MB) were used in the etiological classification process, they were excluded from the regression model to avoid incorporation bias. Five variables were entered into the final model: sex, neutrophil-to-lymphocyte ratio (NLR), HDL-cholesterol, C-reactive protein (CRP), and lactate dehydrogenase (LDH).

The model showed high apparent discrimination in the complete-case dataset [AUC = 0.924, 95% CI (0.887, 0.961)] and adequate calibration (Hosmer-Lemeshow *P* = 0.656). The likelihood ratio test was statistically significant (chi2 = 80.85, *P* < 0.001). However, with only 37 cardiac events among 171 complete cases and 5 predictor variables, the events-per-variable ratio was 7.4, which is below the commonly recommended threshold of 10. Therefore, these results should be considered exploratory.

**Figure 1:**
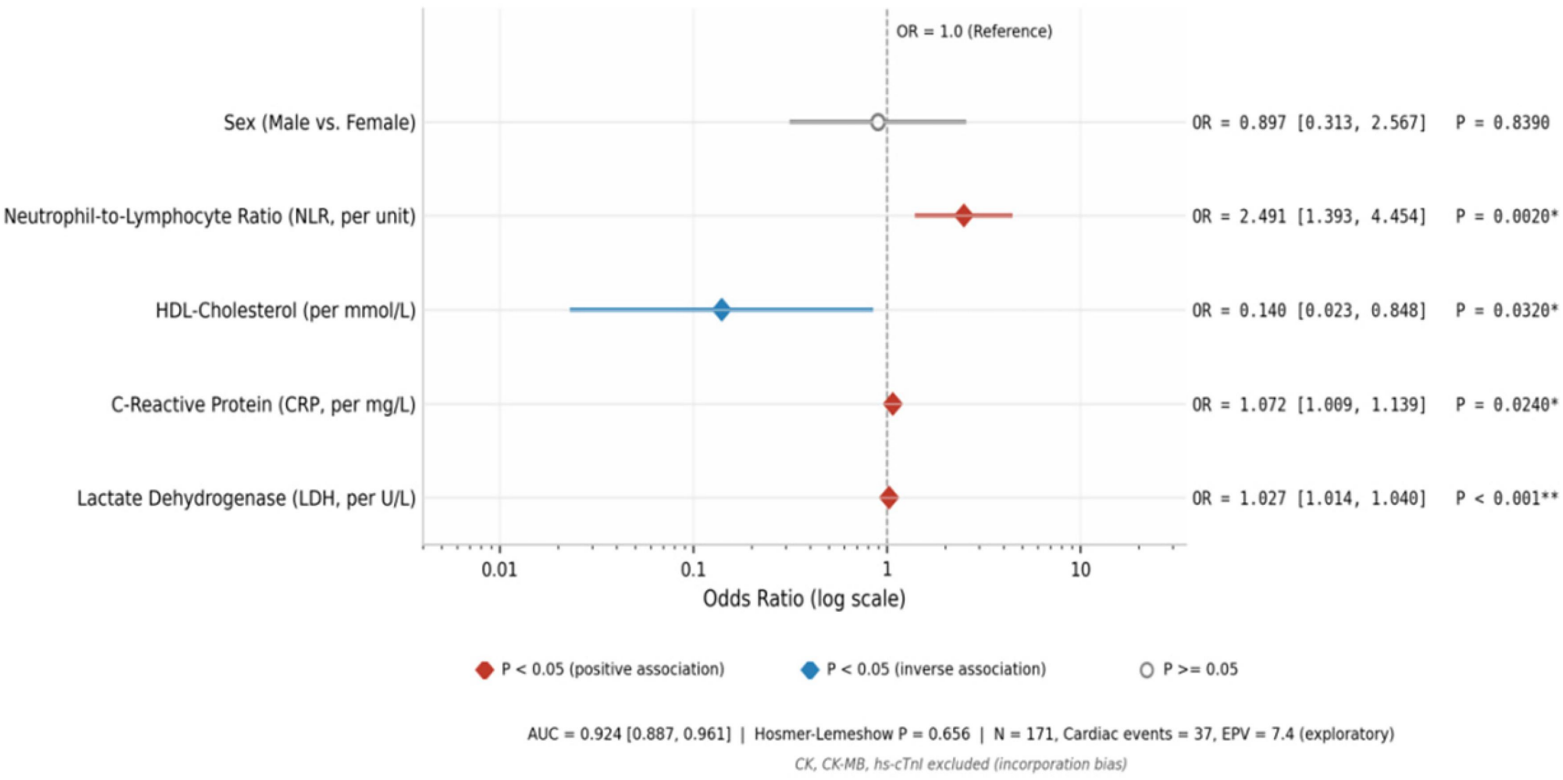
Forest plot of exploratory multivariable logistic regression for factors associated with cardiac chest pain classification. CK, CK-MB, and hs-cTnI were excluded from the model to avoid incorporation bias. NLR, neutrophil-to-lymphocyte ratio; HDL-C, HDL-cholesterol; CRP, C-reactive protein; LDH, lactate dehydrogenase; OR, odds ratio; CI, confidence interval. *P < 0.05, **P < 0.01.

NLR showed the strongest association with cardiac chest pain classification in the exploratory multivariable model [adjusted OR=2.491 per unit increase, 95% CI (1.393, 4.454), *P* = 0.002], followed by LDH [adjusted OR=1.027 per U/L, 95% CI (1.014, 1.040), *P* < 0.001] and CRP [adjusted OR=1.072 per mg/L, 95% CI (1.009, 1.139), *P* = 0.024]. HDL-cholesterol showed an inverse association [adjusted OR=0.140 per mmol/L, 95% CI (0.023, 0.848), *P* = 0.032]. Sex was not significantly associated with cardiac classification after adjustment (OR=0.897, *P* = 0.839). The forest plot is shown in Figure 1.

## 4 Discussion

This study analyzed 243 cases of non-traumatic chest pain in children and adolescents and showed that non-cardiac causes were the most common identified etiologies (47.7%), followed by cardiac causes (20.6%) and unknown causes (31.7%).

Previous studies have also reported that most pediatric chest pain cases are non-cardiac in origin (7, 8). Furthermore, significant differences in clinical characteristics were observed among different etiology groups. Based on international studies (from Saudi Arabia, the United States, and Turkey), we discuss the etiology specificity, diagnostic strategies, clinical management, and study limitations.

It is important to note that some cardiac abnormalities identified in this study, particularly myocardial bridging and simple congenital heart defects (such as atrial septal defect and ventricular septal defect), may represent associated findings rather than definite causes of chest pain. Myocardial bridging was classified as a cardiac cause only when accompanied by objective evidence of ischemia or clinical relevance; isolated myocardial bridging detected incidentally was considered an associated finding. Similarly, congenital defects were classified as cardiac causes only when hemodynamic significance was demonstrated. Therefore, the reported proportion of cardiac chest pain (20.6%) should be interpreted cautiously.

### 4.1 Regional specificity and common features of etiology distribution

#### 4.1.1 Non-cardiac chest pain: global commonality and regional influence

In this study, non-cardiac chest pain accounted for 47.7%, which is consistent with studies from Saudi Arabia (95.5% non-organic causes) (7, 18), the United States (with idiopathic chest pain and gastroesophageal reflux disease as the main causes) (8), and Turkey (48% idiopathic chest pain) (9–11). This supports the observation that benign, non-cardiac causes are common in pediatric chest pain.

Among non-cardiac chest pain cases in this region, respiratory diseases were the most common (63.8%). The relatively high proportion of respiratory diseases may be partly related to local environmental or seasonal factors. However, climate, air quality, and seasonal exposure were not directly measured in this study; therefore, this explanation remains hypothetical. Psychological factors accounted for 15.5% of non-cardiac causes, which is higher than the 7.3% found in Turkey. Academic pressure and family environment may contribute to psychological symptoms in adolescents, but these factors were not directly assessed in this study. This is consistent with Saudi studies, which indicated that parental concern may influence children's anxiety.

Additionally, gastrointestinal diseases (13.0%) and urinary system diseases also accounted for a significant portion, similar to findings in Turkey, where “multi-system diseases can cause nonspecific chest pain (19).” This suggests that a broad differential diagnosis is warranted.

#### 4.1.2 Cardiac chest pain: focusing on high-risk subtypes

The most common cause of cardiac chest pain in this study was coronary artery myocardial bridging (34.0%), followed by large atrial septal defects (16.0%) and supraventricular tachycardia (12.0%). These findings differ from the Turkish study, where mitral valve prolapse was the most common cardiac cause (17). The difference from previous studies may be related to differences in study setting, referral pattern, diagnostic strategy, and classification criteria (16). Regional genetic or lifestyle factors cannot be confirmed by the present study. Although coronary artery myocardial bridging is typically a benign anomaly, 56.0% of patients in this study experienced pain during exertion, and their pain scores were higher. Coronary abnormalities in children require careful evaluation (20). These clinical features may warrant further cardiac evaluation, but they are not specific screening indicators for cardiac chest pain. This finding is consistent with the Saudi study, which noted that palpitations and shortness of breath may suggest a higher risk of heart disease.

It is important to note that this study also included potentially serious diseases such as hypertrophic cardiomyopathy and severe valvular malformations (2.5% of cases). Although the proportion is low, these conditions may progress to heart failure and require long-term follow-up. Previous studies have reported that cardiac chest pain accounts for approximately 5% to 10% of pediatric chest pain cases in emergency department settings (21), and missed diagnoses can have severe consequences, highlighting the need for systematic evaluation (23).

#### 4.1.3 Unknown etiology chest pain: potential causes of diagnostic blind spots

In this study, 31.7% of cases had an unknown etiology, which is higher than studies from Saudi Arabia (4.5%) and Turkey (0%). Possible reasons include:

Limited testing: Routine exercise-induced tests and 24-hour esophageal pH monitoring were not conducted, missing hidden causes such as exercise-induced asthma and gastroesophageal reflux disease.

Self-limited symptoms: 21 cases of chest pain lasted less than 1 minute, and symptoms had already alleviated by the time of the examination.

Hidden psychological factors: Some children exhibited somatic pain without typical anxiety/depression symptoms (12).

Potential under-recognition of musculoskeletal chest pain: Costochondritis, chest wall tenderness, muscle strain, and posture-related pain are among the most common causes of pediatric chest pain in previous studies. Because this was a retrospective study, documentation of chest wall tenderness and musculoskeletal examination may have been incomplete. Therefore, some patients classified as having unknown etiology may actually have had unrecognized musculoskeletal or functional chest pain.

This suggests that clinicians need to combine dynamic follow-up with individualized testing to reduce diagnostic blind spots.

### 4.2 Optimizing diagnostic strategies: evidence-based test selection

#### 4.2.1 Prioritize basic examinations to avoid over-medicalization

The Saudi study showed that 96.5% of children had normal physical exams, and 95.5% had non-specific changes in ECG and chest x-rays, indicating that comprehensive examinations are unnecessary. The U.S. study optimized clinical pathways, reducing the chest x-ray usage rate from 46.1% to 35.6% without missing any diagnoses (22). Based on this study's results, the following patterns were observed:

Low-risk patients (chest pain score <5, pain at rest, no accompanying symptoms) more frequently received ECG and chest x-ray as initial evaluations, whereas coronary CTA was rarely used in this group.

High-risk patients (chest pain score ≥6, pain during exertion, with palpitations/shortness of breath) more frequently underwent myocardial biomarker testing (hs-cTnI, CK-MB) and echocardiography, and exercise ECG or coronary CTA was considered in selected cases.

In this study, myocardial biomarkers hs-cTnI and CK-MB were significantly elevated in the cardiac chest pain group, consistent with the Turkish study. These biomarkers were associated with the cardiac etiology classification in this cohort; however, because they may have been incorporated into the diagnostic process, they should not be interpreted as independent diagnostic predictors.

#### 4.2.2 Targeted expansion of specialized tests to improve diagnostic rate

Given the high incidence of respiratory diseases in this region, pulmonary function tests and respiratory pathogen screening may help evaluate children with cough, fever, or wheezing. For cases with unknown etiology, exercise-induced tests or 24-hour esophageal pH monitoring were infrequently used. These tests have been shown in international studies to have diagnostic value for latent non-cardiac chest pain (13, 15).

For cardiac screening, coronary CTA is highly sensitive to vascular abnormalities but is generally reserved for cases of high suspicion due to radiation risks. Echocardiography is non-invasive and convenient, making it the first choice for structural heart disease. In this study, 38 cardiac chest pain patients had structural abnormalities detected by echocardiography, consistent with the Turkish study, where “echocardiography is the main diagnostic tool for mitral valve prolapse.”

#### 4.2.3 Exploratory multivariable analysis

Exploratory multivariable logistic regression analysis suggested that NLR, HDL-cholesterol, CRP, and LDH were associated with cardiac chest pain classification after adjustment for sex. NLR showed the strongest association in the exploratory model, with each unit increase corresponding to approximately 2.5-fold higher odds of cardiac classification. This finding is biologically plausible, as elevated NLR reflects both neutrophil-driven inflammation and lymphocyte-mediated stress response, which may accompany cardiac conditions such as myocarditis or myocardial ischemia. However, it remains unclear whether this association represents a true pathophysiological marker or merely reflects inflammatory states that co-occurred with cardiac diagnoses.

HDL-cholesterol showed an inverse association with cardiac classification, which may seem counterintuitive given that HDL-C is generally considered cardioprotective. One possible explanation is that children with cardiac conditions in this cohort had different nutritional or metabolic profiles compared with those having non-cardiac causes. Alternatively, this finding may reflect the young age of the study population, in whom traditional cardiovascular risk factor patterns may not yet be established. Caution is warranted in interpreting this association.

It is critical to emphasize the exploratory nature of this multivariable analysis. With an events-per-variable ratio of 7.4 (below the recommended threshold of 10), the model is at risk of overfitting. The wide confidence intervals for HDL-cholesterol (OR 0.140, 95% CI 0.023-0.848) and the non-significant association for sex despite borderline differences in univariate analysis suggest that the sample size was insufficient for stable multivariable estimation. Therefore, these findings should not be used to construct clinical prediction rules or diagnostic algorithms. Rather, they generate hypotheses for validation in larger, prospective cohorts.

### 4.3 Practical significance for clinical management: intervention based on regional characteristics

#### 4.3.1 Etiology-based treatment strategy

Based on the etiology distribution, a “stratified management” approach may be considered:

For benign causes, conservative management such as reassurance, symptomatic pain relief, and psychological support may be considered when clinically appropriate.

Children with respiratory diseases received anti-infection and bronchodilator treatment as clinically indicated.

For children with myocardial bridging, exercise recommendations are typically individualized based on symptoms, objective evidence of ischemia, arrhythmia risk, and pediatric cardiology assessment. Routine restriction of strenuous exercise is not appropriate for all patients with isolated myocardial bridging.

The U.S. study optimized clinical pathways, controlling hospitalization rates at 0.3%, indicating that most children do not require hospitalization. Only high-risk cardiac cases (e.g., hypertrophic cardiomyopathy) require short-term observation, which can help reduce medical costs.

#### 4.3.2 Health education at the family and social level

Studies show that parents’ excessive worry increases children's anxiety, which in turn exacerbates pain. Therefore, parent education, emphasizing that most chest pain is benign, may help prevent psychological burdens for children. For children with psychological-related chest pain, psychological support and school-based mental health education may be beneficial (14).

Physical activity advice should be individualized according to the presence or absence of organic disease and specialist assessment. Children without organic disease generally continued normal activity, while those with cardiac conditions received tailored exercise recommendations based on their specific diagnosis.

### 4.4 Study limitations and future directions

This study is a retrospective study and has the following limitations:

Incomplete data: Some cases with unknown etiology were missed due to incomplete testing, and long-term follow-up to assess prognosis was lacking.

Limited sample representativeness: Only children from the First Affiliated Hospital of Shihezi University were included, excluding cases from community hospitals, which may lead to selection bias.

Insufficient quantification of regional factors: The specific mechanisms of climate, diet, and other factors on etiology were not clarified.

Multivariable model limitations: The exploratory logistic regression model was based on complete cases only (*n* = 171) and had an events-per-variable ratio of 7.4, which is below the recommended threshold of 10. This increases the risk of overfitting and produces wide confidence intervals. Therefore, the model should be interpreted as hypothesis-generating and requires validation in larger prospective cohorts.

Future improvements could include:

Conducting multi-center prospective studies to increase the sample size and improve long-term follow-up.

Quantifying the impact of regional factors, such as comparing etiology distribution across different seasons and altitudes.

Referring to U.S. experience in developing local diagnostic and treatment pathways for chest pain, reducing unnecessary tests and hospitalization rates.

## 5 Conclusion

In this single-center retrospective study of children and adolescents aged 8-17 years with non-traumatic chest pain in the Shihezi area of Xinjiang, non-cardiac causes were the most common identified etiologies, while a substantial proportion of cases remained undetermined. Cardiac diagnoses accounted for 20.6% of cases; however, this relatively high proportion should be interpreted cautiously because some cardiac abnormalities, such as myocardial bridging and congenital heart defects, may represent associated findings rather than definite causes of chest pain.

Exertional chest pain, higher NRS scores, palpitations, shortness of breath, abnormal electrocardiography, and elevated myocardial biomarkers were more frequently observed in patients classified as having cardiac chest pain. Chest x-ray and pulmonary function abnormalities were more frequent in patients with respiratory diagnoses. However, this study did not evaluate diagnostic accuracy, and sensitivity, specificity, predictive values, likelihood ratios, and ROC curves were not calculated. Therefore, these findings should be interpreted as associations rather than independent diagnostic criteria.

Clinical evaluation should rely on careful history taking, physical examination, and risk-based selection of auxiliary tests. Coronary CTA should not be used as a routine screening test for pediatric chest pain and should be reserved for selected patients with appropriate clinical indications. Prospective multicenter studies with standardized diagnostic criteria, systematic assessment of musculoskeletal and psychological causes, and follow-up are needed.

## Data Availability

The original contributions presented in the study are included in the article/Supplementary Material, further inquiries can be directed to the corresponding author.
